# The engagement of cortical areas preceding exogenous vergence eye movements

**DOI:** 10.1371/journal.pone.0198405

**Published:** 2018-06-08

**Authors:** Monika Wojtczak-Kwaśniewska, Anna Przekoracka-Krawczyk, Rob H. J. Van der Lubbe

**Affiliations:** 1 Laboratory of Vision Science and Optometry, Faculty of Physics, Adam Mickiewicz University, Poznań, Poland; 2 Vision and Neuroscience Laboratory, NanoBioMedical Centre, Adam Mickiewicz University, Poznań, Poland; 3 Cognitive Psychology and Ergonomics, University of Twente, Enschede, The Netherlands; Universita degli Studi di Roma La Sapienza, ITALY

## Abstract

Source analyses on event related potentials (ERPs) derived from the electroencephalogram (EEG) were performed to examine the respective roles of cortical areas preceding exogenously triggered saccades, combined convergences, and combined divergences. All eye movements were triggered by the offset of a central fixation light emitting diode (LED) and the onset of a lateral LED at various depths in an otherwise fully darkened room. Our analyses revealed that three source pairs, two located in the frontal lobe–the frontal eye fields (FEF) and an anterior frontal area–, and one located within the occipital cortex, can account for 99.2% of the observed ERPs. Overall, the comparison between source activities revealed the largest activity in the occipital cortex, while no difference in activity between FEF and the anterior frontal area was obtained. For all sources, increased activity was observed for combined vergences, especially combined convergences, relative to saccades. Behavioral results revealed that onset latencies were longest for combined convergences, intermediate for combined divergences, and the shortest for saccades. Together, these findings fit within a perspective in which both occipital and frontal areas play an important role in retinal disparity detection. In the case of saccades and combined divergences stimulus-locked activity was larger than response-locked activity, while no difference between stimulus- and response-locked activity was observed for combined convergences. These findings seem to imply that the electrophysiological activity preceding exogenous eye movements consists of a sensory-related part that is under cortical control, while subcortical structures may be held responsible for final execution.

## Introduction

Eye movements play an integral and crucial role in human vision, as for the detailed perception of objects their respective images have to be properly projected on the fovea. Three types of eye movements are responsible for adjusting our gaze and directing the line of sight to a new object of interest: smooth pursuit, saccades, and vergences [1, 2]. While smooth pursuits hold the image of a moving target on the fovea and saccades bring images of the target onto the fovea, vergences move the eyes in opposite directions to project the images of an object on the fovea of both eyes simultaneously [1]. Pure saccades and pure vergences are rarely observed as separate eye movements in everyday life, since exploring the environment mostly consists of a combination of these eye movements. Although the neural circuitry relevant for pure eye movements, mainly saccades, has been widely explored, the neuroanatomy of combined eye movements is not well understood. In the current study, we intend to demonstrate that reflexive (i.e., exogenous) combined eye movements may involve similar cortical areas as saccades. However, different types of eye movements may involve different cortical areas in varying degrees. Source analyses on event related potentials (ERPs) preceding eye movements, which can be derived from the electroencephalogram (EEG), were carried out to determine the likely involvement of different cortical areas over time.

The functional anatomy underlying the execution of saccades, both reflexive, visually guided (exogenous), and volitional (endogenous) saccades in humans, is well known. Saccades have been extensively studied using various methods, including functional neuroimaging studies (i.e., functional magnetic resonance imaging (fMRI), positron emission tomography (PET), electroencephalography (EEG)), neural stimulation (i.e. transcranial magnetic stimulation (TMS)), lesion studies, and single-neuron recordings. All these methods have greatly contributed to our understanding of the neurophysiology and neuroanatomy of saccades, since they provided complementary data (for reviews see: [3, 4, 5, 6, 7, 8, 9]).

The global view that emerged from the aforementioned studies is the engagement of several subcortical and cortical regions while executing reflexive saccades. The cortical network supporting the generation of reflexive saccade includes: (1) the primary and secondary visual cortices (V1, V2), which are involved in the generation of the saccades, since the stimulation of these regions can elicit saccades [10]; V1 and V2 also mediate the detection of motion and the direction of a moving target [11, 12]; (2) the parietal eye fields (PEF), which disengage fixation and trigger reflexive saccades [13]; (3) the frontal eye fields (FEF), which, like PEF, have a role in fixation disengagement [14], although more recent findings suggest that FEF also accounts for performing accurate saccades [15, 16]; and (4) the supplementary eye fields (SEF), which are located on the border of the supplementary motor area (SMA) and the pre-supplementary motor area (pre-SMA) [17]; the role of the SEF in the execution of saccades was revealed by Tehovnik et al. [18] who demonstrated that stimulation of the SEF can elicit saccades. The research on reflexive saccade-related activity also revealed subcortical regions accounting for the generation of reflexive saccades: (1) the cerebellum; (2) the striatum; (3) the paramedian pontine reticular formation (PPRF); and (4) the superior colliculi (SC). However, given the aim of the present study, which focuses on the cortical network engaged in the preparation and execution of eye movements, the subcortical structures engaged in the preparation of reflexive saccades are only briefly described. The cerebellum accounts for maintaining saccade accuracy [19], the striatum is involved in both saccade initiation and inhibition, while the PPRF encodes monocular commands for saccades. Finally, the SC seems to play a crucial role in fixation control and saccade generation per se. Neggers et al. [20] demonstrated that the SC elicits saccades and modulates the latency of saccades, as larger activity observed in the SC resulted in faster saccades whereas another study showed that the inactivation of the SC caused deficits in saccade target selection [21].

Interestingly, Schiller et al. [10, 22] proposed that two processing streams–anterior and posterior–control reflexive saccades. The anterior stream includes FEF and SEF, and reaches the brainstem directly while bypassing SC, whereas the posterior stream includes the parietal and occipital cortex and accesses the brainstem through the SC. This division also relates to the different functional roles that these streams play, since the posterior stream is thought to mediate the generation of saccades, while the anterior system would select the target of the saccade.

In everyday life, we regularly perform vergences, as we constantly have to direct our gaze into different depths. A proper visual exploration regularly requires performing combined eye movements, in which both saccadic and vergence components can be distinguished. Even though vergences are essential for 3D perception, the involved neural pathways, both cortical and subcortical, are unclear as research on vergences is scarce. Two types of cues are well known for their capability to elicit vergences: the blur of an image, and binocular retinal disparity. The latter is considered to be a crucial factor for triggering vergences [1]. The earliest studies on vergences focused on brain areas that process information about retinal disparity. Disparity-tuned cells have been found in V1 and V2 [23], and in subcortical (superficial layers of SC) [24] areas of a cats brain. Likewise, disparity detectors were also identified in the visual cortices (V1, V2, and V3) of primates [25, 26, 27]. Moreover, micro stimulation and single-neuron studies [28] showed that the rostral part of the SC generates information that accurately keeps the eyes in 3D position.

A recent study on humans using TMS [29] also demonstrated that the FEF are engaged in the execution of memory guided (i.e., endogenous) vergences. However, the results of EEG and fMRI studies indicated that a region of the frontal cortex located anterior to the saccade-related FEF may be involved in both volitional [30] and reflexive [31] vergences. Moreover, Gamlin and Yoon (2000) revealed that activation of an area anterior to the FEF (including the area between the arcuate sulcus and the posterior pole of the principal sulcus) is related to motoric aspects of vergence preparation, but not to sensory aspects, i.e. the detection of retinal disparity.

Studies using neuroimaging methods like PET and fMRI imply a high spatial resolution, but also a low temporal resolution. As a consequence, the transient activity of specific brain areas can easily be missed. Furthermore, the temporal sequence of the involved cortical brain areas remains rather obscure. Due to its high temporal resolution, EEG may provide important features of the temporal dynamics of the variously involved cortical mechanisms. According to our knowledge, only two EEG studies on vergences in humans have been published [32, 33]. Both studies explored the cortical mechanisms associated with reflexive eye movements: pure saccades, pure vergences, and combined vergences. These studies employed different paradigms and analyzed their data in a different way. Kapoula et al. (2002) investigated response-locked brain activity just before eye movement execution. More negativity was observed preceding pure divergences than preceding pure convergences. Similarly, for combined divergences more negativity was observed than for combined convergences. Methodological restrictions (a small number of participants, a limited number of electrodes, and few trials per condition) did not allow them to determine the cortical topography associated with vergences. Nevertheless, their findings suggest that frontal and parietal areas are involved preceding the execution of vergences. In turn, Tzelepi et al. (2004) examined stimulus-locked brain activity after the onset of a relevant stimulus, so they focused on the sensory aspects of eye movement control. They observed that convergences were preceded by more negativity, which seemed more related to the N1 component, than divergences. Although both studies investigated cortical activity related to vergences, their results cannot easily be compared. Stimulus-locked analyses in the study of Tzelepi et al. (2014) predominantly reveals the processing of the stimulus that elicits eye movements, while response-locked analyses, as reported in the study of Kapoula et al. (2002) emphasizes the activity related to the execution of eye movements. Furthermore, the results of these studies can also not easily be considered as complementary, since the experimental design and setup were not the same.

In the current study, we tried to determine the cortical areas related to the execution of exogenous vergences, and additionally attempted to specify the possible roles of these areas. As a comparison of ERP topographies related to the execution of the different eye movements may be quite cumbersome and may not lead to clear conclusions we thought it might be more useful to describe the ERPs in terms of the activities of different underlying cortical sources. We used the BESA (brain electrical source algorithm) method, which enables to describe ERPs by different cortical sources with different activity patterns over time. The outcome of these analyses may also provide relevant information about the possible interplay between different cortical areas. Initially, we compared a response-locked activity for the different eye movement types as this may clarify whether a specific area is more relevant for a specific type of eye movement. Secondly, we compared a response-locked and stimulus-locked activity. The differences between these activities may indicate whether the underlying process is more related to eye movement execution or to processing the target of the eye movement.

## Material and methods

### Participants

Sixteen healthy volunteers (12 females and 4 males) with an average age of 22.6 years (SD = 0.7) participated in this study. Two of them were excluded from the analyses since in both cases over 40% of their eye movements were accompanied by blinks, which makes a proper assessment of eye movement onset problematic. All participants reported being right-handed. None of the participants had a history of any neurological or psychiatric disorders.

The study was approved by the local ethics committee of the Adam Mickiewicz University and was performed in accordance with the Declaration of Helsinki. Written informed consent was obtained from all of the participants.

### Optometric examinations

All participants took part in an optometric examination session. The administered test measured refractive errors and monocular distance and near visual acuity (Snellen’s letter chart) with corrected refractive errors. Subsequently, the following parameters of binocular vision were measured: (1) distance and near heterophoria using a cover test with a prism bar, (2) fusional vergence ranges using a prism bar, (3) suppression using a Worth 4-dot test and a Pola Mirror test, (4) the near point of convergence (NPC) measured by a push-up method (5) and stereopsis, by using the Paul Harris Randot Test (Stereo Optical).

Optometric eye examinations revealed that the visual acuity of both eyes of participant’s eyes was in a normal range for near and far space (logMAR ≤ 0.00). In some cases, refractive errors were corrected by glasses or contact lenses (participants with contact lenses were included only when they used this correction on a daily basis). None of the subjects presented any suppression and all achieved at least 30 sec of arc in the stereopsis test. Far heterophoria ranged from 2 prism diopters of exophoria to orthophoria, whereas near heterophoria ranged from 6 prism diopters of exophoria to 1 prism diopters of esophoria. The averaged values of the fusional vergence ranges and near point of convergence (break and recovery) are listed in Table 1, whereas measured parameters for each participant are presented in S1 Table.

**Table 1:** Averaged clinical parameters of optometric examination for positive (base-out) and negative (base in) fusional range, the break and recovery point of near point of convergence.

Parameter	Averaged values	
**Positive fusional range at far (prdptr)**	18.7 ± 4.8	
**Negative fusional range at far (prdptr)**	8.5 ± 1.4	
**Positive fusional range at near (prdptr)**	26.6 ± 6.2	
**Negative fusional range at near (prdptr)**	16.1 ± 3.2	
**Near point of convergence–break (cm)**	3.4 ± 1.7	
**Near point of convergence–recovery (cm)**	4.8 ± 1.9	

### Task and stimuli

**Fig 1:**
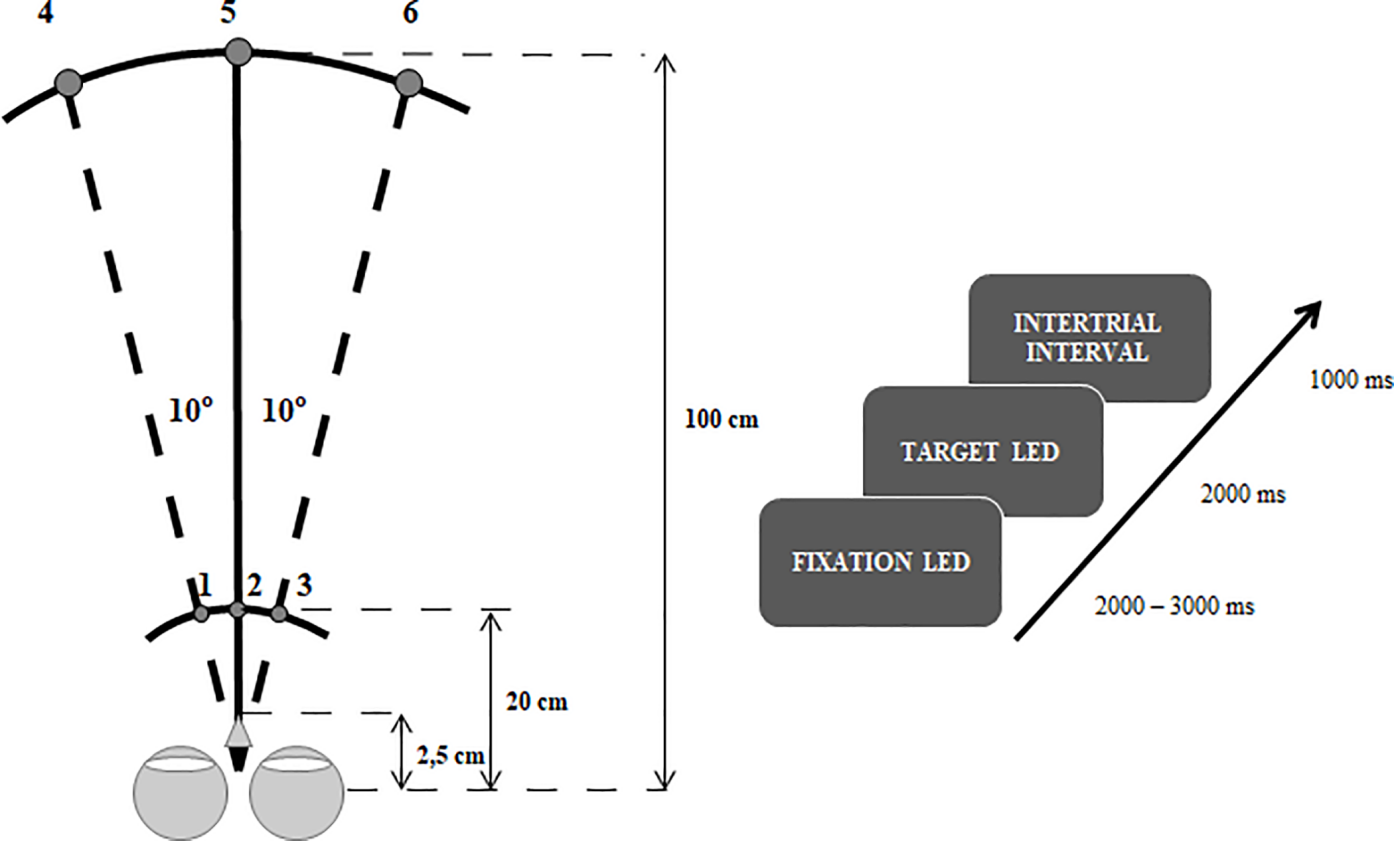
**A) A graphical representation of the experimental setup.** LEDs (light-emitting diodes) were placed at eye level on isovergent circles at two distances from the observer: 20 cm and 100 cm. Eye movements were elicited for both distances, depending on the combination of the fixation and the target LED. Each trial started with the onset of the fixation LED in the midline (position 2 or 5) which was followed by the onset of one of the lateral target LEDs. Eye movements were triggered by redirecting the eyes as following: saccades at far–from LED 5 to LED 4 (leftward saccades) or 6 (rightward saccades); saccades at near–from LED 2 to LED 1 (leftward saccades) or 3 (rightward saccades); combined convergences–from LED 5 to LED 1 (leftward combined convergences) or 3 (rightward combined convergences); combined divergences–from LED 2 to LED 4 (leftward combined divergences) or 6 (rightward combined divergences). **B) The design of the experiment as a function of time.** Each trial started with the fixation LED, displayed for a random duration between 2,000 to 3,000 Subsequently the target LED was presented for 2,000 ms, which was followed by a 1,000-ms intertrial interval.

All stimuli were displayed on a device with LEDs located at various positions (see Fig 1). Six LEDs were positioned at the eye level on isovergent circles at far and near visual space: 1 m and 20 cm from the center of eye rotation (located 2.5 cm from the nasal bridge), respectively. The lateral separation of LEDs was 10°, so the stimuli triggering pure saccades produced zero retinal disparity, whereas stimuli triggering both vergence eye movements produced 12° disparity (both crossed and uncrossed). Moreover, the lateral separation was adjusted and based on a previous study [34] which showed that below 15° saccades do not require head movements and are more natural. The chin and forehead were stabilized to exclude head movements, which would disrupt EEG and electrooculographic (EOG) signals. Three main types of eye movement were elicited: pure saccades for far and near positions, combined convergences, and combined divergences. Each type of eye movement to the right and to the left LED had to be carried out 51 times, resulting in a total of 408 trials in the experiment. The three blocks of 136 trials, which were presented in a counterbalanced order, were separated by ten-minute breaks. The experiment was preceded by a demo block, which consisted of all the eight different eye movement types, which were repeated five times and were presented in a random order. Eye movements (EOG signal) were monitored online to ensure that participants performed the task in line with the instructions.

Each trial started with the onset of the fixation LED at the near or far position in the midline (LED 2 or 5 in Fig 1A). The fixation LED was presented for a random duration between 2,000 to 3,000 ms in 50 ms steps to reduce temporal anticipation. The fixation LED was followed by the onset of one of the lateral target LEDs which was displayed for 2,000 ms. The design of the experiment in the function of time is presented in Fig 1B.

The experiment was carried out in a completely darkened room. Participants could only see the LED that they had to fixate on. The brightness of the LEDs was controlled and individually adjusted so that the near and the far LEDs were perceived as equally bright. The size of the LEDs was also adjusted depending on their distance: near LEDs (0.3 cm) were smaller than far LEDs (1.2 cm) in such a way that they stimulated a similar size of the retina.

Before the experiment started, verbal instructions were given. Participants were instructed to make an eye movement from the fixation LED to the target LED as quickly but also as accurately as possible. On average, the experimental part took approximately one hour per participant.

### EEG recordings

The EEG was registered from 64 active electrodes placed on an actiCap (Brain Products GmbH) located on positions according to the extended International 10–20 system [35]. A ground electrode was affixed at AFz. An average reference was used. Electrode resistance was kept below 5 kΩ. The signal was amplified by a QuickAmp 128 amplifier (Brain Products GmbH) with a sample rate of 500 Hz, and was filtered online with a low cut-off filter of 0.015 Hz to remove slow drifts possibly related to small head movements.

**Fig 2:**
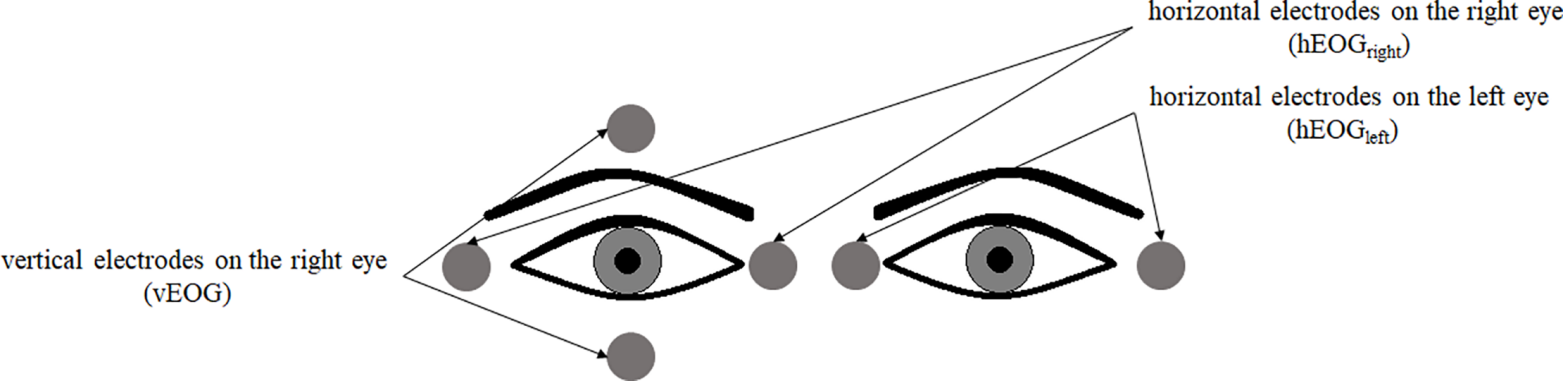
Electrode placement for EOG data collection. First, the saccade signal was registered separately for left and right eyes. Subsequently, they were averaged using the following formula: EOG_sacc_ = (hEOG_left_ + hEOG_right_)/2.

Eye movements and blinks were recorded by measuring the EOG from three bipolar electrodes (Fig 2): one of them was located above and below the right eye (for vertical eye movement and blink detection–vEOG) and two other electrodes were mounted on the outer and inner canthi of both the right and the left eyes (for horizontal eye movement detection–hEOG_right_, hEOG_left_). The EOG was filtered with low cut-off (0.25 Hz) and high cut-off filters (30 Hz). EEG, EOG, and digital markers signaling relevant events in the experiment were registered with a Brain Vision Recorder (Brain Products GmbH). The EOG and EEG signals were analyzed offline with Brain Vision Analyzer 2.0.3 (Brain Products GmbH) software.

### Behavioral analyses

Every eye movement elicited in this study contains a saccadic component. The onset and peak of the registered eye movement were marked on the saccade signal (EOG_sacc_). The saccade signal was initially determined using the following formula: EOG_sacc_ = (hEOG_left_ + hEOG_right_)/2, to eliminate differences between the left and right eye [36]. The onset and peak of each eye movement was determined on a trial by trial basis using the EMG onset search algorithm (implemented in the Brain Vision Analyzer version 2.0.4.). The onset of an eye movement has been defined as follows: the mean amplitude and its standard deviation are calculated for a baseline period (0–100 ms). The onset criterion is subsequently set to a quintuple of the SD above or below the mean, depending on its polarity. Thus, the implemented solution searches for the time point at which EMG activity exceeds 5 SD from the mean of the baseline period. Trials with improper eye movements, which was based on an additional thorough visual inspection, were excluded from further analyses. Based on the determined eye movement onset, latencies can be defined as the time interval from stimulus onset to eye movement onset. The following exclusion criteria were used: incorrect direction, premature onset (< 100ms), and too slow onset (> 600ms). We chose the latter criterion as eye movements slower than 600 ms appear not to be exogenously triggered. Eye movements accompanied or preceded by eye blinks were additionally excluded. In total, the number of removed segments amounted to 9.40%.

### EEG analyses

Segments from -400 to 400 ms relative to eye movement onset were extracted from the raw EEG data, which were large enough to be able to perform both response-locked and stimulus-locked analyses. After application of the behavioral exclusion criteria, an artifact rejection procedure was performed: trials with major artifacts were excluded from any further analyses (maximum allowed voltage step: 100 μV/ms, lowest allowed activity within 50 ms intervals: 0.1 μV). Subsequently, we corrected for artifacts of non-cortical sources (e.g., muscle artifacts, electrocardiac artifacts etc.) by employing the semiautomatic Independent Component Analysis (ICA) algorithm. The average number of removed components amounted to 1.8. After application of the ICA procedure, the artifact rejection procedure was repeated with more strict criteria (maximum allowed voltage step: 50 μV/ms, minimum/maximum allowed amplitude: ±150 μV, maximum allowed difference of values within 200 ms intervals: 200 μV). The artifact rejection procedures carried out before and after ICA resulted in the removal of less than 1% of the data. Subsequently, averages were computed for each condition and each electrode.

To minimize the possibility that the observed activity reflects eye movement execution, we restricted our analyses to a -180 to -60 ms time interval. In this time interval, motoric aspects of eye movement preparation were investigated. The selected interval was based on the longest observed average latency (179 ms for leftward combined convergences, see the Results section). Including earlier time windows might reflect activity related to the fixation LED, which appeared before the target LED. To enable a comparison of the response- and stimulus-locked activity, an appropriate time interval had to be selected for the stimulus-locked analyses. To avoid interference from subsequent eye movements, we based our selection on the latency of the saccades, which had the shortest latencies (see the Results section). This latency (135 ms) determined the last analyzed time interval (120–140 ms relative to the stimulus). The length of the time interval for stimulus-locked analyses was chosen to be the same as for the response-locked analyses, i.e. 120 ms. Therefore, we selected the time interval from 20 to 140 ms after stimulus onset.

### Source analysis method

BESA software (version 6.0, MEGIS Software GmbH) was used to localize the cortical generators related to the preparation and execution of the different eye movement types. All 64 channels were included in the analyses. The cortical activation underlying the ERPs may be described by current dipoles [37] or by regional sources [38, 39]. We decided to use regional sources as they have been reported to create more stable models [40].

The locations of the regional sources of the observed scalp potentials were determined for the time range from -180 to -60 ms before eye movement onset using the BESA algorithm. A PCA of the grand averages per condition revealed that three symmetrical regional source pairs should be sufficient to describe 99.2% of the data. The global field power (GFP) of the grand average was used to identify relevant time windows, which were selected from the onset of the peak of the GFP to its local maximum. The fitting procedure was sequentially applied for the relevant time windows, and one or more symmetrical source pairs were inserted to describe activity in each time window. The aim of the fitting procedure was to find a best-fit solution that minimizes the residual variance (RV). We assumed that the same areas are involved in all eye movement types but that these areas are involved in different degrees. Initially, a model was built and based on the ERPs averaged across all types of eye movements and participants. One symmetrical source pair was fitted to each of the relevant time windows i.e. (1) -180 to -154 ms, (2) -130 to -60 ms. This model described the preparation of saccades and combined divergences quite well, resulting in the low values of RV. However, it seemed insufficient to properly describe combined convergences (RV = 8%). Adding a second source pair in each relevant time window resulted in a decrease of RV. However, we intended to obtain a similarly low RV value with three pairs of regional sources for all conditions, since a PCA of the grand averages showed that a model with three regional pairs should be sufficient to describe the observed data. Therefore, we decided to determine the regional sources for the grand averages of combined convergence. Here, three relevant time windows were identified: (1) -180 to -154 ms, (2) -132 to -96 ms, and (3) -86 to -60 ms. One symmetrical source pair was fitted to each time interval, resulting in a significant reduction of the RV. Adding more sources did not further reduce RV. As a final check, we also examined a model based on fMRI data from a study by Alkan et al. (2011) [31]. Even though this model included a larger number of sources it did not result in a solution with lower RV, so it is obviously less parsimonious. Based on these examinations, we decided to use the model with three regional sources.

Source activities were estimated by applying the selected model on the ERPs for each type of eye movement per participant [41, 42]. Subsequently, the root-mean-square (RMS) values for each separate source was determined, which creates an estimate of overall source activity [40]. This procedure was applied for each participant, and each type of eye movement. The acquired data was subsequently used for the statistical analyses.

### Statistical analyses

For the statistical analyses, STATISTICA 12 software was used. The onset latencies of the eye movements were evaluated by an analysis of variances (ANOVAs) with repeated measures with two factors: *eye movement type* (saccades, combined convergences, and combined divergences) and *direction* of the eye movement (right or left).

**Fig 3:**
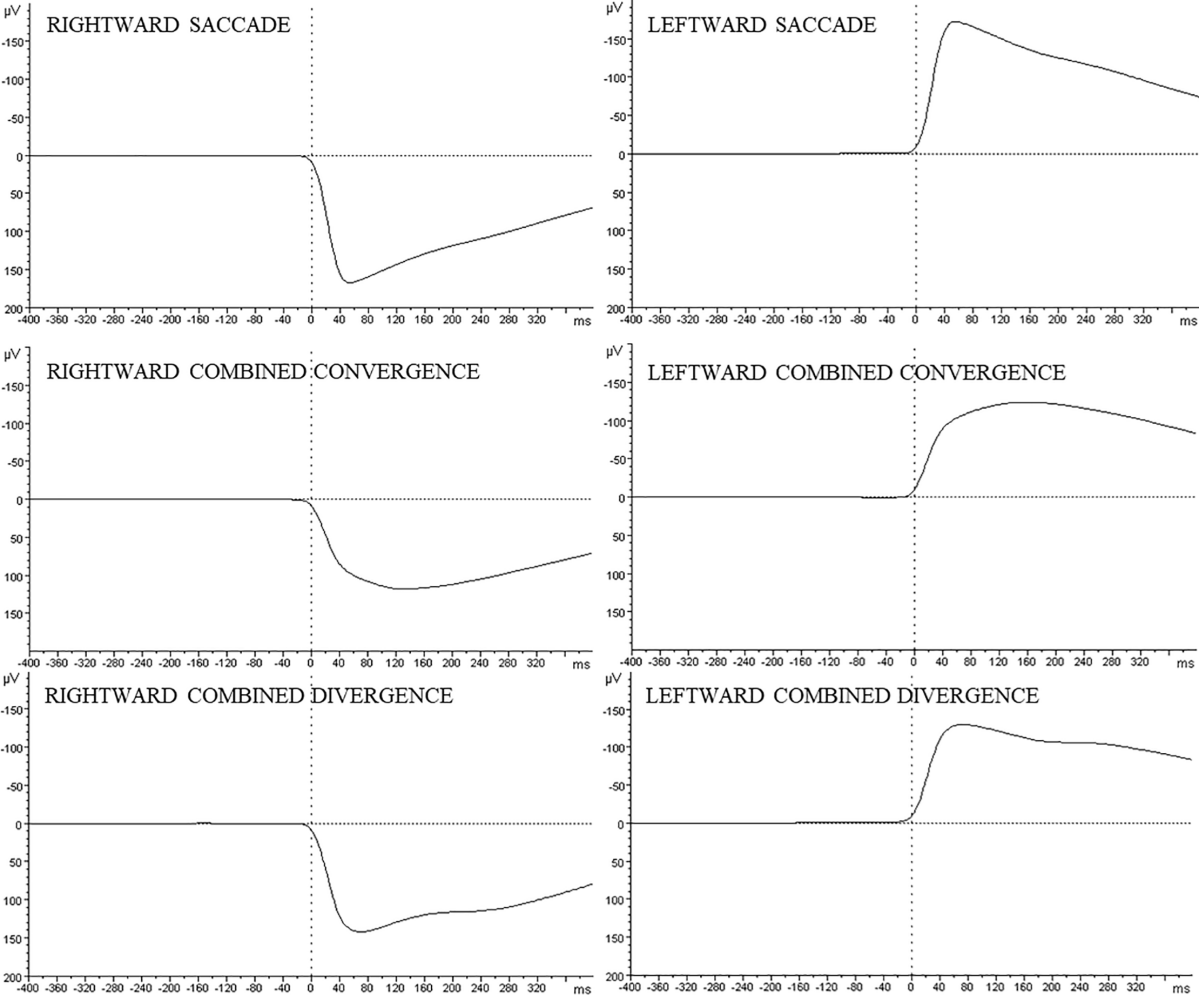
Electro-oculographic (EOG) signals measured from the electrodes attached to the outer and inner canthi of both the right and the left eyes. The grand averages, based on 14 participants, are presented for each condition from -400 to 400 ms relative to the saccade onset of each eye movement.

The statistical analyses of RMS for a response-locked activity were carried out on 20-ms intervals from -180 to -60 ms before saccade onset to examine the preparation of the different exogenous eye movement types (Fig 3). Although we removed all trials with detectable premature eye movements, to check whether observed cortical activity is not due to eye movement execution the dependency between EOG and the estimated source activities was examined. We used Pearson’s correlation coefficient, *r*, and correlated the estimated source activities with the EOG. For saccades we used EOG_sacc_ (determined above), while for vergences we used EOG_verg_ (calculated using the following formula: EOG_verg_ = (hEOG_left_−hEOG_right_)). Since in the case of vergences, the eyes move in opposite directions, we calculated the difference between horizontal movements of the left and right eye rather than the average.

The statistical analyses of response-locked source activities were carried out by using a repeated measures ANOVA with the following factors: *source* (RS1, RS2, RS3), *time* (six 20-ms time windows starting from -180 ms to -60 ms relative to eye movement onset), *eye movement type* (saccades, combined convergences and combined divergences), *direction* (to the right or to the left), and *hemisphere* in which the source was located (left or right). However, to enable an interpretation of several complex significant interactions, we also performed ANOVAs for each regional source separately.

To compare response-locked and stimulus-locked data, a repeated measures ANOVA was performed with the factors: *time* (six 20-ms time windows), *event* (stimulus- or response-locked), and *eye movement type* (saccades, combined convergences and combined divergences). To reduce the complexity of the analyses, we computed averages across rightward and leftward eye movements, which were subjected to the analyses.

To be able to properly interpret possible activity differences within the most posterior source, we additionally compared stimulus-locked contralateral activities between eye movement types for this source. To calculate the contralateral activity, activity after right LEDs within left and activity after left LEDs within right posterior sources were averaged. Different activities for each condition shortly after stimulus onset (at 100 ms) might imply that initial processing already depends on the information to be extracted. The statistical analyses included the following factors: *time* (six 20-ms time windows starting from 20 ms to 140 ms relative to stimulus onset), and *eye movement type* (saccades, combined convergences and combined divergences).

For all of the employed analyses, significance was assigned at the p ≤ 0.05 level. ANOVAs were followed by Tukey’s posthoc tests, and Huynh–Feldt ε correction was performed when necessary.

## Results

### Behavioral data

Eye movement onset latencies are presented in Table 2. Saccades were characterized by the shortest mean onset latencies, intermediate latencies were obtained for combined divergences, and the longest latencies were observed for combined convergences. ANOVA confirmed the presence of a major effect of *eye movement type* (*F*(2,26) = 75.2, *p* < 0.001, *η*^*2*^ = 0.65). Post-hoc tests revealed that saccades were faster than combined divergences (*p* < 0.001); and combined divergences were faster than combined convergences (*p* < 0.001). The analysis revealed that latencies were not dependent on the direction of the eye movements, since there was no main effect of *direction* (*F*(1,13) < 0.01, *p* = 0.953, *η*^*2*^ < 0.001), and also no *eye movement type* x *direction* interaction (*F*(2,26) = 0.02, *p* = 0.943, *η*^*2*^ = 0.001).

**Table 2:** Mean latencies for exogenously triggered saccades, divergences, and convergences based on 14 participants. SE represents the standard error.

Eye movement type		Latency [ms]	SE [ms]
**Saccades**	Rightward	136	5
	Leftward	135	6
**Divergences**	Rightward	154	5
	Leftward	153	5
**Convergences**	Rightward	178	6
	Leftward	179	8

Source analyses revealed that the following cortical areas are strongly related to the execution of the different eye movement types (for details see the Source analysis section): (RS1) an anterior frontal area, (RS2) the occipital cortex, and (RS3) the FEF.

The analyses showed that in the -100 to -80 ms time interval, for RS1 significant correlations were observed for rightward combined convergence between right RS1 and EOG_verg_ (*p* = 0.001), and left RS1 and EOG_verg_ (*p* = 0.003). Significant correlations were observed also for leftward combined convergence between right RS1 and EOG_verg_ (*p* = 0.045). In the next time interval -80 to -60 ms significant correlations were observed for rightward combined convergence between right RS1 and EOG_verg_ (*p* = 0.032). For sources located within the left and right RS2 no significant correlation was observed in both -100 to -80 ms (EOG_sacc_ *p* ≥ 0.137, EOG_verg_ *p* ≥ 0.108) and -80 to -60 ms time intervals (EOG_sacc_ *p* ≥ 0.082, EOG_verg_ *p* ≥ 0.283). For RS3 a significant correlation was observed in the -100 to -80 ms time interval, whereas in the subsequent time window no significant correlation was observed (EOG_sacc_ *p* ≥ 0.062, EOG_verg_ *p* ≥ 0.084).

Based on these results, it may be concluded that the activity of RS1 reflects a residual eye movement artifact (see Discussion), whereas correlations observed for RS3 seem due to chance effects as no consistent pattern of correlations was observed over time. Specifically, if these correlations were induced by eye movements, similar correlations should have been observed for subsequent time windows.

### Event related potentials

**Fig 4:**
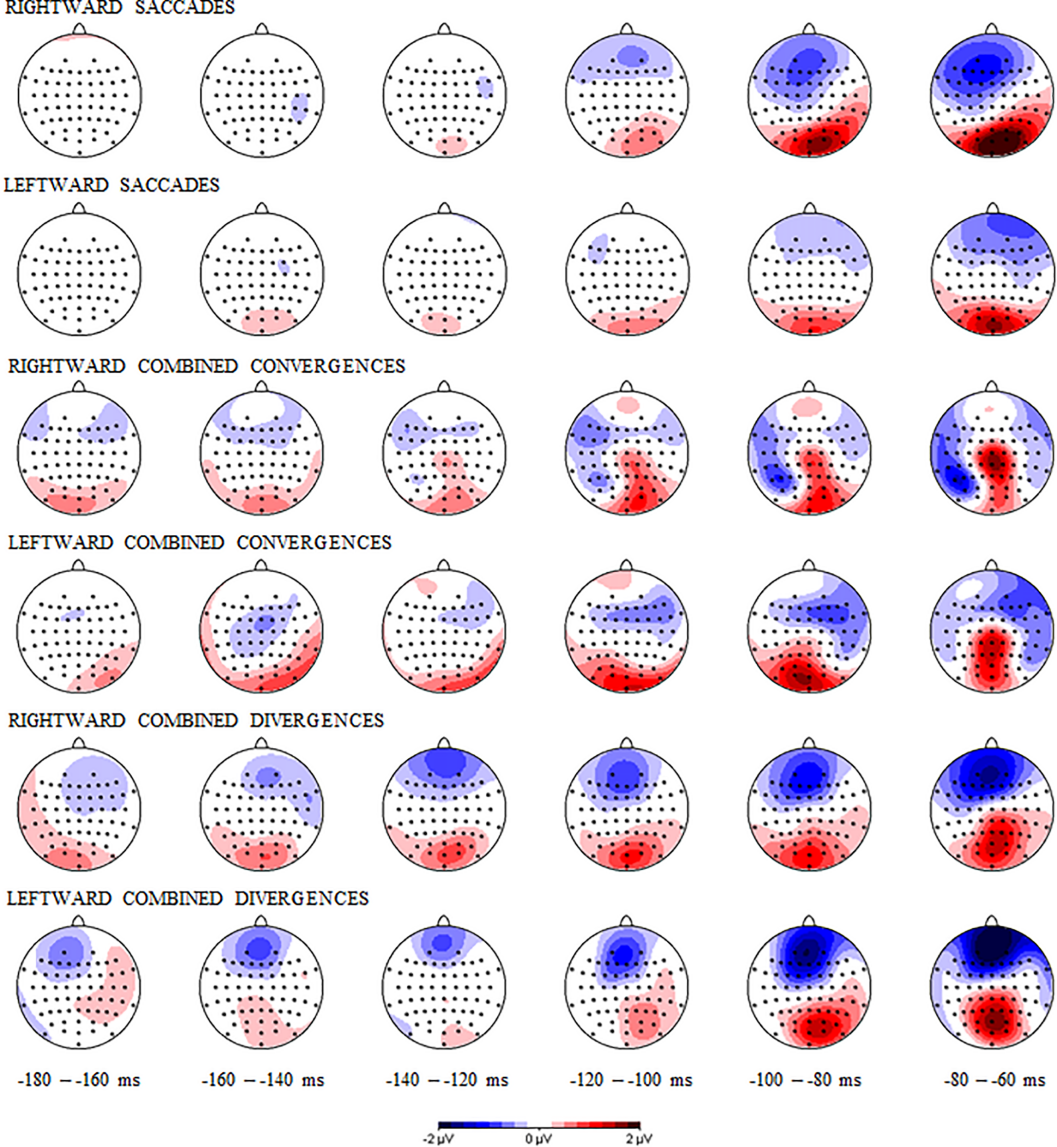
Topographical maps of event-related potentials for the three eye movement types from -180 ms to -60 ms before eye movement onset. The first two rows display the maps for pure left and right saccades, the third and fourth row show the maps for combined left and right convergences, and the two lower rows represent the maps for left and right combined divergences.

**Fig 5:**
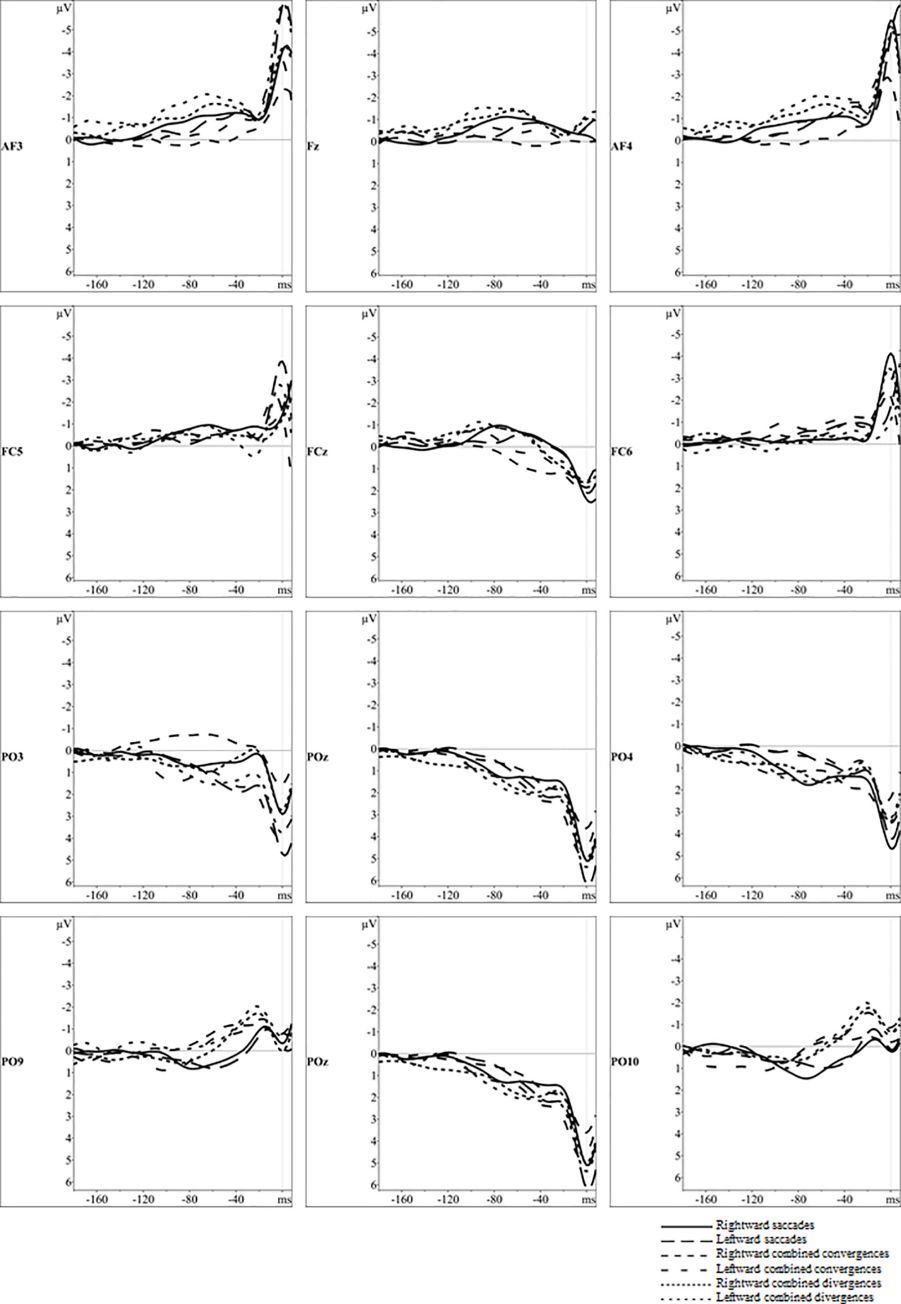
Grand average waveforms of event related potentials for three eye movement types in time intervals from -180 ms to 10 ms relative to eye movement onset. **The** grand averages waveforms of the response-locked activity are presented for 12 relevant electrodes located in the frontal, parieto-occipital and occipital cortex.

The topographical maps of the grand averages of ERPs showing cortical activity from -180 to -60 ms relative to eye movement onset for the different eye movement types are displayed in Fig 4, whereas the grand average waveforms of ERPs for representative electrodes are shown in Fig 5. As indicated above, we focused on the source analysis results and used the grand averages of ERPs to prepare the model.

### Source activities

**Fig 6:**
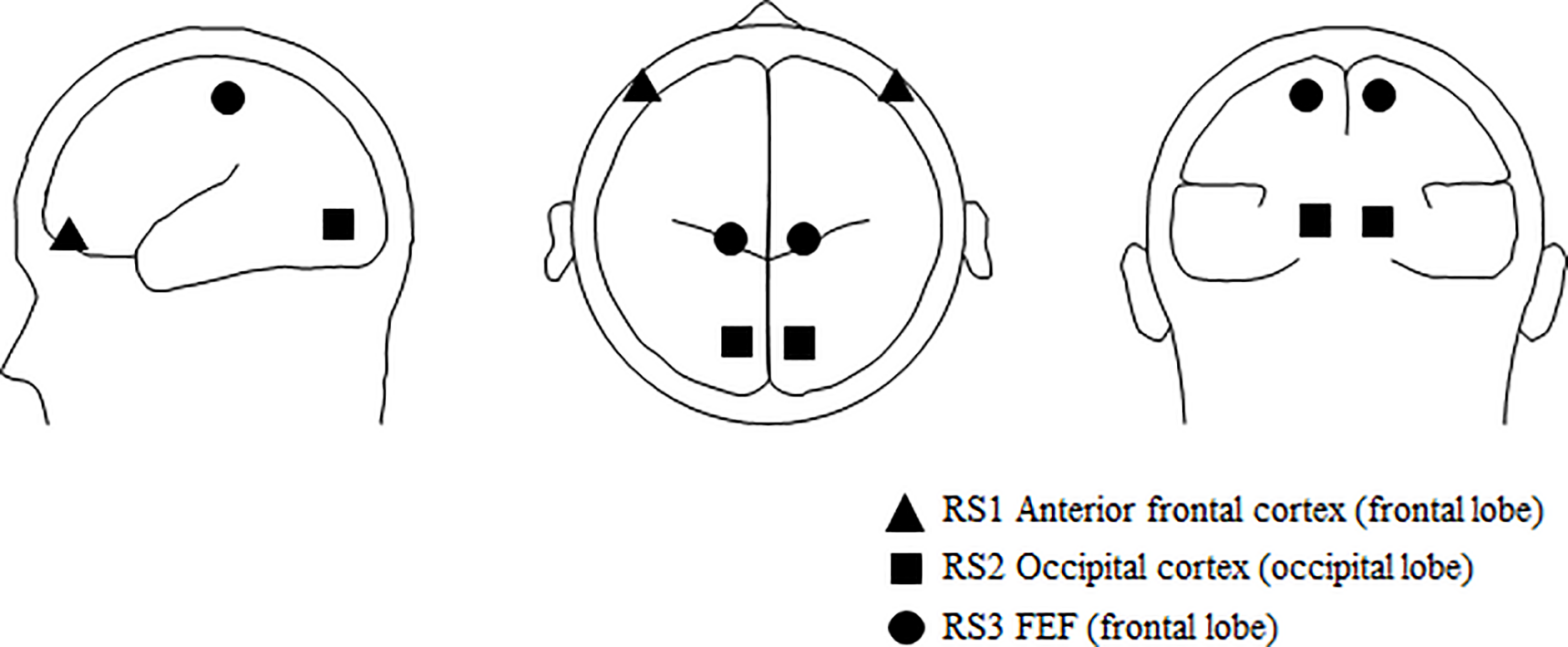
Estimated pairs of source locations based on event-related potentials (ERPs) observed preceding lateral exogenous saccades, convergences, and divergences. A model was built based on the averaged ERPs obtained for combined convergences using the BESA algorithm. Based on the GFP inspection, the fitting procedure was performed for the three time windows selected from the onset of a peak to the local maximum of the GFP: (1) -180 to -154 ms, (2) -132 to -96 ms, and (3) -86 to -60 ms. Three pairs of regional sources (RS) were fitted to subsequent time intervals: RS1, RS2, and RS3, respectively.

A PCA on the obtained grand averages indicated that RV may be substantially reduced, up to 1.81%, when using three regional sources. Based on inspection of the GFP we decided that the fitting procedure should be performed for the following three time windows selected from the onset of a peak to the local maximum of the GFP. Each regional source (RS) pair was fitted to a subsequent time interval, i.e. RS1 to the -180 to -154 ms time window, RS2 to the -132 to -96 ms time window and RS3 to the -86 to -60 ms time window. In Fig 6, a model is presented displaying the estimated locations of the different regional source pairs.

The obtained RV amounted to 1.56% for the saccade condition, 4.82% for the combined convergence condition, and 2.78% for the combined divergence condition. The regional sources that seem related with the execution of the different eye movement types are located in: (RS1) an anterior frontal area (the frontal lobe), (RS2) the occipital cortex (the occipital lobe), and (RS3) the FEF (the frontal lobe). Precise locations of the regional sources, in terms of Brodmann areas and Talairach-Tournoux coordinates, are indicated in Table 3. Anatomical regions were determined using the Talairach Client software (version 2.4.3), using Single Point Search [43]. As mentioned in the Materials and Methods section, we also examined models with a different number of sources, however, the chosen model with three pairs of symmetrical regional sources described activity preceding the eye movements in the most optimal way, as an increase in the number of sources did not substantially reduce RV and a reduction in the number of symmetrical sources substantially increased RV (>8%).

**Table 3:** Talairach coordinates of regional sources related to the execution of saccades, convergences, and divergences. The regional sources were determined for the time window -180 ms to -60 ms before the eye movement onset. A model was built and based on the averaged ERPs obtained for combined convergences using the BESA algorithm. Cortical regions were estimated using the Talairach Client software (version 2.4.3).

Regional sources	Fitting time intervals	Cortical region	Brodmann Area	X	Y	Z
**RS1**	-180 to -154 ms	Anterior frontal cortex	**10**	**+/-53**	**57**	**0**
**RS2**	-132 to -96 ms	Occipital cortex	**18**	**+/-12**	**-81**	**-5**
**RS3**	-86 to -60 ms	Frontal eye field	**6**	**+/-13**	**-31**	**55**

**Fig 7:**
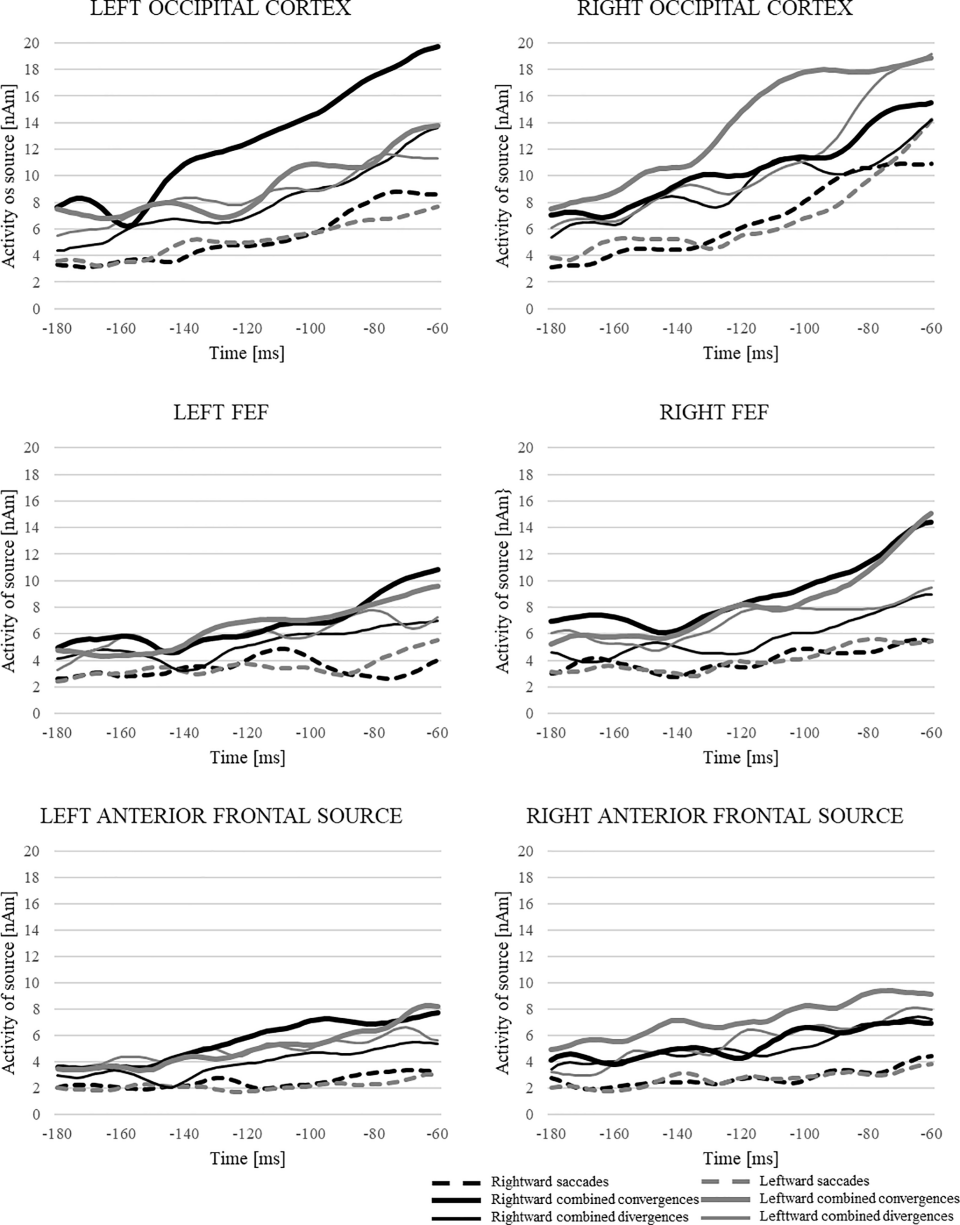
Source waveforms elicited by activity preceding the different eye movement types for each regional source. Source waveforms were prepared per participant by calculating the RMS (root mean square) for each condition within each of the three regional sources per hemisphere. Then, the averages of all individuals per condition were computed, the result of which is shown in the figure. The obtained individual averages for each type of eye movement per condition were used for statistical analyses.

Overall, we observed a significant main effect of *source* (*F*(2,26) = 23.6, *p* < 0.001 *η*^*2*^ = 0.64). Post-hoc tests revealed that activity was larger in RS2 than in RS3 (*p* < 0.001) and larger in RS2 than in RS1 (*p* < 0.001). No difference in activity was observed between RS1 and RS3 (*p* = 0.13). The analyses performed for each regional source separately showed that activity differed over time, being largest shortly before eye movement onset for RS2 (main effect of *time*, for RS2 (*F*(6,65) = 30.6, *p* < 0.001, *η*^*2*^ = 0.70)), for RS3 (main effect of *time* (*F*(6,65) = 29.2, *p* < 0.001, *η*^*2*^ = 0.69)) and for RS1 (main effect of *time* (*F*(6,65) = 15.8, *p* < 0.001, *η*^*2*^ = 0.55)). A contrast analysis revealed a linear trend (for RS2 (*F*(1,13) = 37.1, *p* < 0.001), for RS3 (*F*(1,13) = 42.8, *p* < 0.001)) and for RS1 (*F*(1,13) = 19.1, *p* < 0.001)). Fig 7 suggests that these effects reflected a general increase in source activity. Source activity was also larger for right hemispheric than for left hemispheric sources for RS2 (main effect of *hemisphere* (*F*(1,13) = 7.1, *p* = 0.02, *η*^*2*^ = 0.35)), RS3 (main effect of *hemisphere* (*F*(1,13) = 15.1, *p* = 0.002, *η*^*2*^ = 0.54)) and RS1 (main effect of *hemisphere* (*F*(1,13) = 9, *p* = 0.01, *η*^*2*^ = 0.41)).

#### Occipital cortex (RS2)

For RS2, analysis of the source activities revealed a main effect of *eye movement type*, *F*(2,26) = 30.1, *p* < 0.001, *η*^2^ = 0.70). This effect concerned differences between all *eye movement types*: saccades vs. combined convergences (*p* < 0.001), saccades vs. combined divergences (*p* < 0.001), and combined convergences vs. combined divergences (*p* = 0.007). As can be noticed in Fig 7, the largest activity was observed prior to combined convergences, intermediate prior to combined divergences, and the lowest prior to saccades. Additionally, a significant interaction between *direction* and *hemisphere* (*F*(1,13) = 5.3, *p* = 0.04, *η*^*2*^ = 0.29) was observed. Post-hoc tests revealed that the hemispherical differences only concerned leftward eye movements, which elicited the strongest activity in the right hemisphere (*p* = 0.02). We did not observe similar results for rightward eye movements (*p* > 0.05).

A significant interaction between *eye movement type*, *direction*, and *hemisphere* (*F*(2,26) = 11.1, *p* = 0.002, *η*^*2*^ = 0.46) was observed. Furthermore, interaction between *eye movement type*, *direction*, *hemisphere* and *time* was found (*F*(10,130) = 3.6, *p* = 0.002, *η*^*2*^ = 0.21). To enable an interpretation of these complex interactions, we performed ANOVAs for each type of eye movement.

For saccades the interaction between *direction* and *hemisphere* (*F*(1,13) = 0.03, *p* = 0.875, *η*^*2*^ < 0.01) as well as the interaction between *direction*, *hemisphere* and *time* was insignificant (*F*(5,65) = 2.4, *p* = 0.082, *η*^*2*^ = 0.15). For combined divergences the interaction between *direction* and *hemisphere* was also insignificant (F(1,13) = 0.6, *p* = 0.448, *η*^*2*^ = 0.04), but the interaction of these factors with *time* was in turn significant (*F*(5,65) = 4, *p* = 0.012, *η*^*2*^ = 0.24). Post-hoc tests revealed that differences only concerned the last time window (-80 to -60ms), where within the right hemisphere rightward combined divergence was related to higher activity compared to leftward combined divergence (*p* < 0.001), and rightward combined divergence evoked higher activity in the right hemisphere, compared to the left hemisphere (*p* < 0.001). In turn, for combined convergences both interactions were significant (*direction* * *hemisphere* (*F*(1,13) = 9.7, *p* = 0.008, *η*^*2*^ = 0.43); *direction*, *hemisphere* * *time* (*F*(5,65) = 5.1, *p* = 0.005, *η*^*2*^ = 0.28)). Post-hoc tests performed for the *direction* and *hemisphere* interaction revealed that these differences were related to leftward combined convergences which evoked higher activity in the right RS2 compared with the left RS2 (*p* = 0.042). Moreover, the post-hoc test performed for *direction*, *hemisphere* and *time* interaction revealed that leftward combined convergence was related to higher activity in the right hemisphere (*p* < 0.039) in the time window -140 to -60 ms. Post-hoc tests also revealed that in the time window -120 to -80 ms within right RS2 leftward combined convergences evoked higher activity compared to rightward combined convergences (*p* < 0.005) and conversely–within left RS2 in time window -100 to -60 ms rightward combined convergences evoked higher activity compared to leftward combined convergences (*p* < 0.02).

#### Frontal eye field (RS3)

For RS3, a main effect of *eye movement type* was observed (*F*(2,26) = 41.5, *p* < 0.001, *η*^*2*^ = 0.76). We observed differences between all conditions: saccades vs. combined convergences (*p* < 0.001), saccades vs. combined divergences (*p* < 0.002), and combined convergences vs. combined divergences (*p* < 0.002). The activity was the largest for combined convergences, lower for combined divergences, and lowest for saccades. The interaction between *eye movement type* and *time* revealed that the differences as a function of eye movement type was not constant (*F*(2,26) = 41.5, *p* < 0.001, *η*^*2*^ = 0.76). The post-hoc tests revealed that a statistically significant difference between saccades and combined convergences (*p* < 0.001) was observed for all time intervals (-180 to -60 ms). In turn, the significant differences between saccades and combined divergences were only observed in the time window from -140 to -60 ms (*p* < 0.001), and the differences between the two combined vergence conditions were only observed shortly before eye movement onset (-100 to -60 ms) (*p* ≤ 0.027).

#### Anterior frontal cortex (RS1)

Separate statistical analysis performed for RS1 showed significant differences in the level of activation between conditions (*F*(2,26) = 9, *p* = 0.006, *η*^*2*^ = 0.41). The largest activity was observed for combined convergences and the smallest activity was found for saccades. Post-hoc tests disclosed that in the analyzed -180 to—60 ms time window, activity of the source for combined convergence was larger than for saccades (*p* = 0.001), and activity for combined divergence was significantly larger than for saccades (*p* = 0.018). No differences were observed between both vergence conditions (*p* = 0.479). Here, we also observed that the direction of the eye movements influence the level of activity (*F*(1,13) = 8.2, *p* = 0.013, *η*^*2*^ = 0.39). It was higher for left eye movements than for right eye movements (*p* = 0.013).

When the influence of the hemisphere was taken into account, no differences in the activity between eye movement types were observed (*direction* * *hemisphere* interaction (*F*(1,13) = 3.3, *p* = 0.092, *η*^*2*^ = 0.20). Moreover, the differences between eye movements were not dependent on the direction of the eye movements (eye movement type * direction interaction *F*(2,26) = 1.5, *p* = 0.246, *η*^*2*^ = 0.10) as well as time (eye movement type * time interaction, *F*(10,130) = 1.4, *p* = 0.275, *η*^*2*^ = 0.09).

Separate analyses per eye movement type revealed that in RS1 the effect of lateralization was only observed for combined convergences (significant direction * hemisphere interaction *F*(1,13) = 11.9, *p* = 0.004, *η*^*2*^ = 0.48). Post-hoc tests revealed that this lateralization effect was limited to leftward combined convergences, which elicited larger activity compared to the rightward combined convergence on the right hemisphere (*p* = 0.011), and to the leftward combined convergence which elicited higher activity in the right hemisphere compared to the left hemisphere (*p* = 0.002).

### A comparison of response-locked and stimulus-locked activities

**Fig 8:**
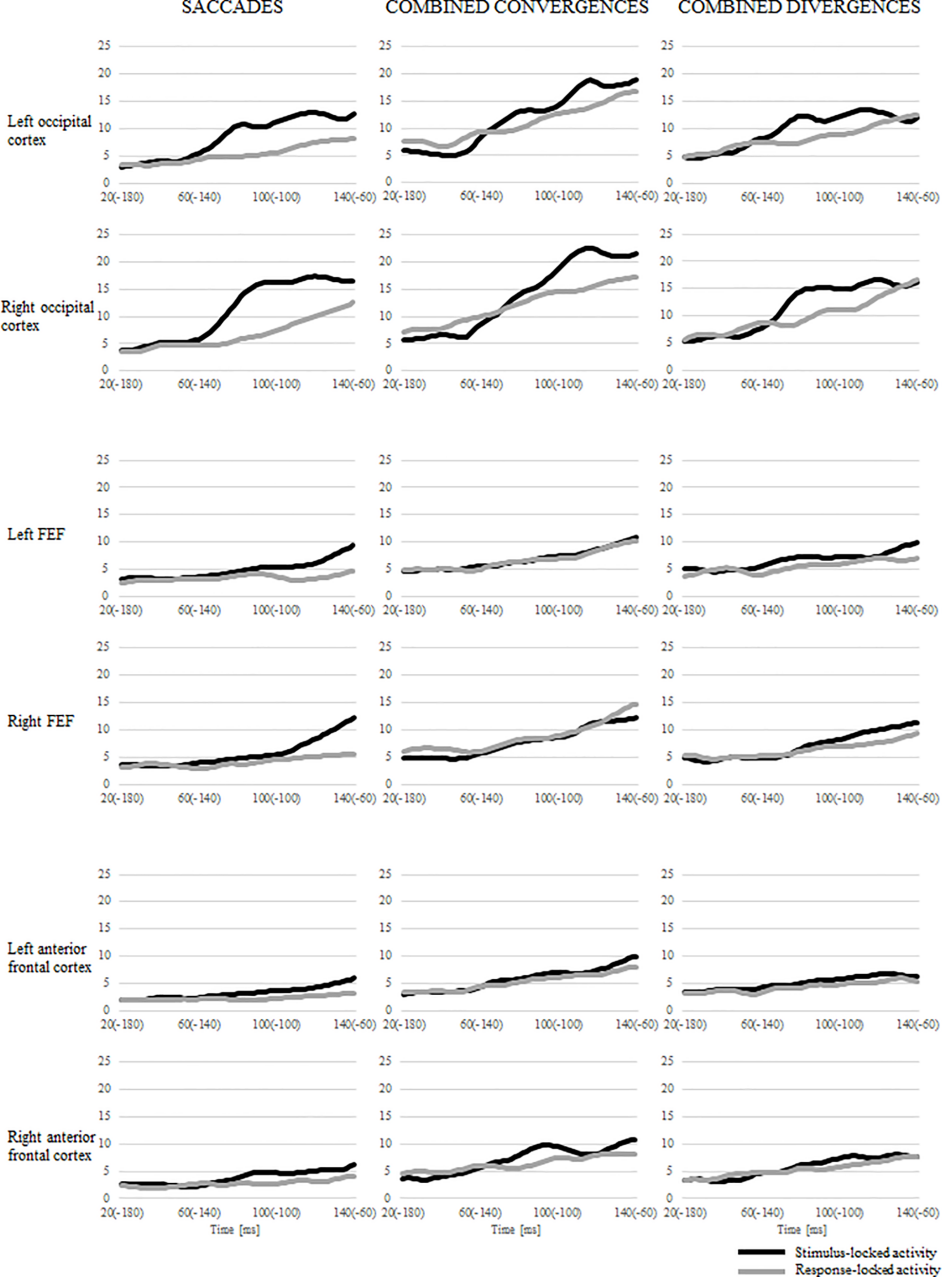
Source waveforms comparing stimulus-locked and response-locked activities in each regional source for saccades, combined convergences and combined divergences.

To be able to better interpret the observed source activities, we compared response-locked activity from -180 to -60 ms with stimulus-locked activity from 20 to 140 ms relative to target stimulus onset. In Fig 8, a comparison of the response- and stimulus-locked activity is presented for saccades, combined convergences, and combined divergences for each regional source.

Within left and right RS2, a significant preponderance of stimulus-locked activity for saccades was demonstrated (main effect of *event* (*F*(5, 65) ≥ 53.6, *p* < 0.001, *η*^*2*^ ≥ 0.8). Moreover, a significant interaction between *time* and *event* revealed that this preponderance concerned the time interval from 60–140 ms after stimulus onset (-140–-60 ms before eye movement onset) (*F*(5, 65) ≥ 8.5, *p* < 0.001, *η*^*2*^ ≥ 0.39). For combined divergences also a main effect of *event* was observed (*F*(5, 65) ≥ 7.9, *p* ≤ 0.015, *η*^*2*^ ≥ 0.38). The preponderance of the stimulus-locked activity concerned the 60–120 ms time interval for left RS2 (*time* * *event*, *F*(5, 65) = 5.8, *p* < 0.001, *η*^*2*^ = 0.31) and the 80–120 ms time interval for right RS2 (*time* * *event*, *F*(5, 65) = 4.2, *p* = 0.011, *η*^*2*^ = 0.24). Within the RS2 we did not observe differences between the stimulus- and response-locked activity for combined convergences (main effect of *event F*(1, 13) ≤ 3.1, *p* ≥ 0.1, *η*^*2*^ ≤ 0.19), but significant interaction between *time* and *event* for the right RS2 (*F*(5, 65) = 6.18, *p* = 0.002, *η*^*2*^ = 0.32) was observed. A post-hoc test revealed that the stimulus-locked activity was higher than the response-locked activity, which concerned the -100 to -80 ms time window (100–120 ms after the stimulus onset) (*p* = 0.001).

Within both the right and left RS3, for saccades a higher stimulus-locked than response-locked activity was observed (main effect of *event F*(1,13) ≥ 17, *p* ≤ 0.001, *η*^*2*^ ≥ 0.57) which concerned the 100–140 ms time interval (*time* *event, *F*(5, 65) ≥ 9.0, *p* < 0.001, *η*^*2*^ ≥ 0.41). For combined divergences a significant main effect of *event* was observed only for the left RS3 and stimulus-locked activity was higher than the response-locked (*F*(1, 13) ≥ 10.9, *p* = 0.006, *η*^*2*^ ≥ 0.46), whereas for the right RS3 the differences between the stimulus-locked and response-locked activities were not significant (main effect of *event F*(1, 13) = 2.7, *p* = 0.124, *η*^*2*^ = 0.17). However, the significant interaction between *time* and *event* (*F*(5, 65) = 3.3, *p* = 0.033, *η*^*2*^ = 0.2) revealed that in the last time window 120–140 ms differences between the stimulus-locked and response-locked activity was significant showing a preponderance of a stimulus related activity. For combined convergences no differences between stimulus- and response-locked activities were observed (main effect of *event F*(1, 13) ≤ 1.7, *p* ≥ 0.212, *η*^*2*^ ≤ 0.12)

Activity was larger within the left and right RS1 stimulus-related activity than the response-related activity for saccades (main effect of *event F*(1, 13) ≥ 34.9, *p* < 0.001, *η*^*2*^ ≥ 0.73) and it was related mainly to the 80–140 ms time window (*time* * *event*, *F*(5, 65) ≥ 3.7, *p* ≤ 0.071, *η*^*2*^ ≥ 0.32). For combined divergences we only observed overall differences between the stimulus- and response-locked activities (main effect of *event F*(1, 13) ≥ 4.9, *p* ≤ 0.044, *η*^*2*^ ≥ 0.28) with a preponderance of stimulus-related activity. For the left RS1, no significant differences for combined divergences between stimulus-locked and response-locked activities over time were observed (*time* * *event*, *F*(5, 65) = 0.6, *p* = 0.686, *η*^*2*^ = 0.04). In turn, for the right RS1 the interaction between *time* and *event* was significant, (*F*(5, 65) = 3.3, *p* = 0.024, *η*^*2*^ = 0.2), however post-hoc tests showed that it did not concern any type of eye movement (*p* > 0.1). No difference between stimulus- and response-locked activity was observed for combined convergences (main effect of *event F*(1, 13) ≤ 4.6, *p* ≥ 0.051, *η*^*2*^ ≤ 0.26). However, we observed a significant interaction between *time* and *event* for the right RS1 (*F*(5, 65) = 3.8, *p* = 0.01, *η*^*2*^ = 0.23). The stimulus-locked activity was higher than the response-locked activity and the post-hoc test showed that it was observed in the -120 to -100 ms time window (80–100 ms after the stimulus onset) (*p* = 0.012).

Overall, the stimulus-locked activity was larger than the response-locked activity in the case of saccades and combined divergences, especially within RS2 concerning a time interval of about 100 ms after stimulus onset. Within RS1 and RS3 still the overall preponderance of activity related to stimuli was observed for saccades and combined divergences. However, for saccades we observed similar results with the enhanced preponderance of the stimulus-locked activity in 80–140 time interval, but for combined divergence there was no difference between stimulus related and response related activities in any time window. For combined convergences no differences between the stimulus- and response-locked activities were observed.

**Fig 9:**
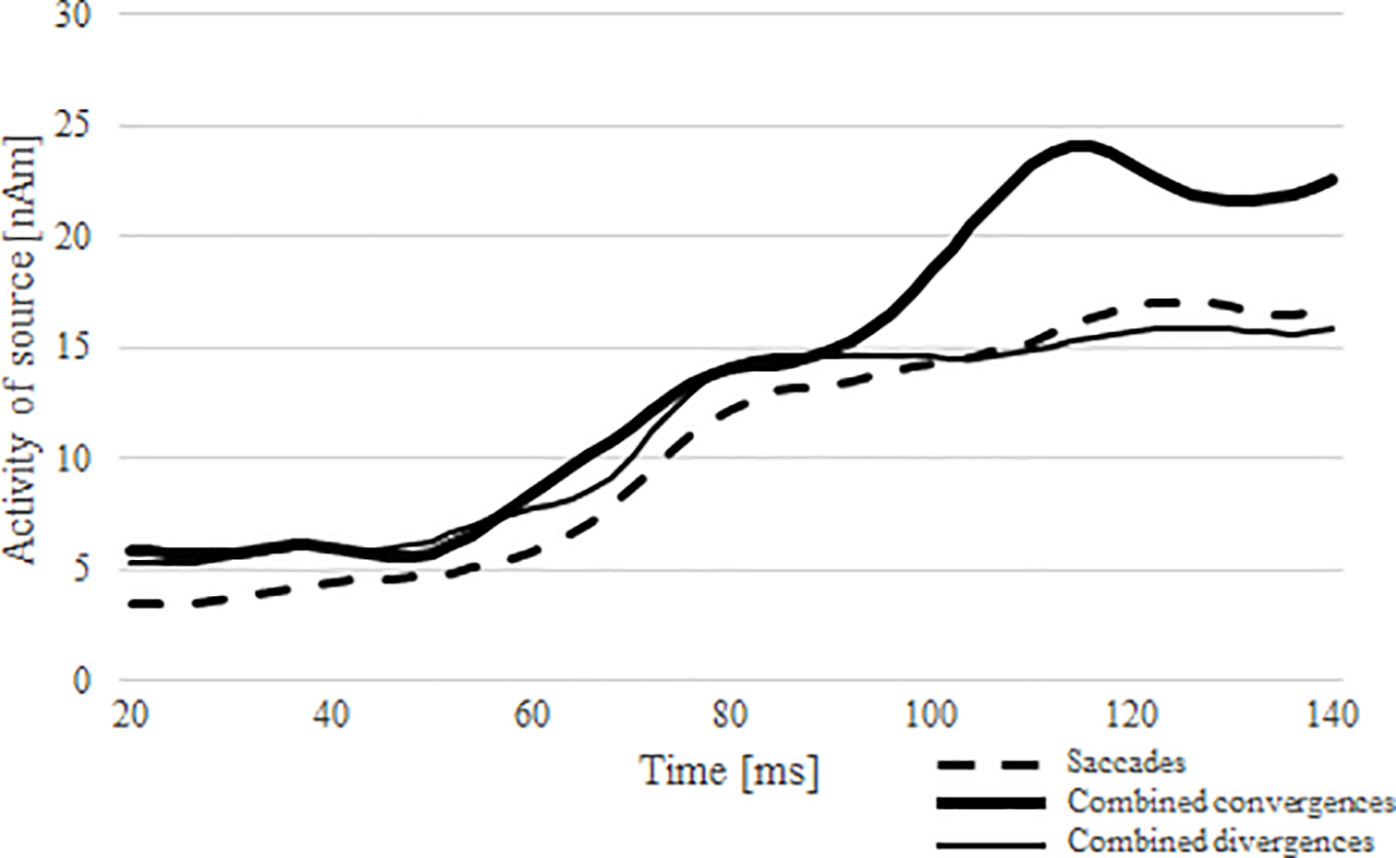
Contralateral source activity elicited by eye movement preparation in occipital source RS2. Source waveforms were prepared per participant by calculating the RMS (root mean square) for each condition within a regional source. Then, the averages of all individuals per condition and contralateral activity were computed. Finally, the result is shown in the figure. The obtained averages for each type of eye movement were used for statistical analyses.

Finally, we compared a contralateral stimulus-locked activity for the three types of eye movement within RS2 (Fig 9). Here we calculated the contralateral activity for saccades, combined convergences and combined divergences (i.e., averaged activity which evoked rightward eye movement within the left RS2 and leftward eye movements within the right RS2). An ANOVA with two factors was performed: *time* (6), and *eye movement type* (3). Comparable activities for all conditions would suggest that activity solely depends on stimulus processing while differences between conditions might support the involvement of additional processes like the detection of crossed or uncrossed disparity. Interestingly, within the occipital cortex a significant main effect of *eye movement type* was observed (*F*(2,26) = 8.5, *p* = 0.004, *η*^*2*^ = 0.40) and post-hoc tests revealed that combined convergences were related to higher activity as compared to saccades (*p* = 0.002) and combined divergences (*p* = 0.014) as well. Moreover, the interaction of *time* and *eye movement type* (*F*(10,130) = 2.8, *p* = 0.038, *η*^*2*^ = 0.18) and a post-hoc test revealed that the differences between conditions concerned the time windows from 100 ms till 140 ms after stimulus onset (*p* < 0.008).No differences were observed between saccades and combined divergences (*p* = 0.64).

### The influence of far and near stimuli for cortical activity within occipital source RS2

In the present study, we also asked whether differences between saccades and vergences (mainly combined convergences and divergences) are possibly due to differences in perception between near and far LEDs, e.g. higher illumination of LEDs presented at near, and thus higher stimulation of the retina and or the size of LEDs. To answer this question, we directly compared the stimulus-locked activity within RS2 between saccades performed at both near and far distances as similar activation for both types of saccades ensures that differences between different eye movement types are not due to the illumination of different LEDs. We performed a repeated measures ANOVA with the following factors: *time* (six 20-ms time windows starting from 20 ms to 140 ms relative to stimulus onset), and *distance* (near and far). Here, results indicated that occipital activity was independent of distance (main effect of *distance*, (*F*(1,13) < 0.1, *p* = 0.891, *η*^*2*^ < 0.01). We observed a significant interaction between *time* and *distance* (*F*(1,13) = 3, *p* = 0.047, *η*^*2*^ = 0.17), however, post-hoc tests revealed that in none of our time windows a difference was observed between near and far saccades (*p* > 0.254).

## Discussion

In the present study, we specifically addressed the question whether the brain areas thought to be relevant for the execution of exogenously triggered eye movements, as identified with the source analysis, are differentially involved depending on the type of eye movement (i.e., saccades, combined convergences, and combined divergences). We also intended to improve our understanding of the observed activities of these brain areas. For example, we would like to know whether the observed activities are more strongly related to eye movement execution, or to specific perceptual processes that are required to perform the different eye movement types.

Saccades were characterized by the shortest latencies, intermediate latencies were observed for combined divergences, while the longest latencies were observed for combined convergences. These results replicate previous findings [33, 44, 45], although some other studies observed the longest latencies for divergences [46, 47]. In general, it can be concluded that vergences are characterized by longer latencies compared to saccades [48]. This seems to imply that the preparation of exogenously triggered combined vergences engage additional processes (e.g., attentional and/or visuomotoric) needed to carry out these eye movements. This could be associated with disparity detection, which differentiates saccades from vergence eye movements. Furthermore, discrepancies in the latencies between the vergence conditions may be related to the detection of different types of binocular disparity (the differences in the location of the stimuli on the retina between the right and left eye). The latency differences seem related to crossed disparity in the case of combined convergences and uncrossed disparity in the case of combined divergences.

The main goal of the present study was to determine and understand the role of different cortical areas for the execution of natural eye movements that involve a depth component. Analyses with BESA on the grand averages on ERPs revealed that three pairs of regional sources may account for the observed ERPs within the -180–60 ms time interval. Based on Talairach Client software (version 2.4.3), we estimated that these source pairs are located within the occipital cortex, FEF, and an anterior frontal area, which correspond to RS2, RS3, and RS1 respectively. Based on the observed correlations between EOG and the anterior frontal sources, and on the vicinity of the left and right orbits the activity of the anterior frontal source was interpreted as a residual eye movement artifact that was not fully excluded with the ICA procedure.

Our results revealed that significantly the largest response-locked activity was related to the occipital cortex and no differences were observed between both frontal areas. These results may be related to the primary and crucial role of the occipital cortex, associated with the projection to higher-order cortical areas [49, 50], which triggers neuronal circuits engaged in the execution of eye movements. This hypothesis strongly corresponds to the fitting procedure, in which the occipital cortex was fitted to the second and FEF to the third time interval. Altogether, these findings may reflect the temporal sequence of the engagement of the indicated cortical areas. Moreover, regardless of the source location, combined convergences generally induced a larger response-locked activity compared to saccades and combined divergences. Since our results show a general preponderance of stimulus-locked as compared to response-locked activity this suggests that the estimated source activities are related to sensory aspects preceding eye movement execution.

We additionally noticed that irrespective of the direction of the executed eye movement, source activity in the right hemisphere was always larger than in the left hemisphere, and this effect was observed for all regional sources. These observations seem in line with the view that spatial attention especially concerns the right hemisphere as it deals both with left and right external space while the left hemisphere seems only related to right external space [51]. An earlier study with saccades also reported this same asymmetry [52], which was related to spatial attention [52, 53]. Furthermore, our results are also in accordance with studies on hemispatial neglect [54], as problems with spatial attention are often associated with right hemispheric lesions within both occipital and parietal regions.

Although the determination of the engaged cortical sources was one of the major aims of the present study, quite crucial and interesting conclusions may be derived from the comparison of response-locked and stimulus-locked activity source activities. This analysis allows for a more detailed interpretation of the observed source activities. A preponderance of a response-locked activity would suggest that the activity relates to motoric aspects of eye movement generation, whereas the preponderance of stimulus-locked would point to sensory-related processing of the eye movement inducing stimuli. Our results revealed a preponderance of stimulus-related activity for saccades and combined divergences, and no difference between stimulus-related and response-related activities for combined convergences. The overall lack of a preponderance of response-locked activity, but general preponderance of stimulus-locked activity indicates that the activity of the determined cortical sources is strongly related to stimulus processing. Our results seem to be partially in line with the above-mentioned division indicated for saccades by Schiller et al [10, 22]. They suggest that an anterior stream including FEF is related to target processing, whereas a posterior stream including subcortical areas mediate eye movement execution.

The occipital cortex has obviously a crucial role in the processing of various aspects of visual stimuli [50, 49, 55, 56, 57, 58]. Our results demonstrate that a stimulus-locked activity is in general larger than a response-locked activity, which confirms the view that the engagement of the visual cortex in our study is more related to stimulus processing than to eye movement execution. Interestingly, the preponderance of stimulus-locked activity compared to response-locked was particularly evident at about 100 ms after stimulus presentation which may be associated with the posterior P1 component elicited by visual stimuli [59, 60]. Importantly, our results additionally revealed a greater response of the occipital cortex for combined convergences and combined divergences as compared to saccades. This observation seems to imply that perceptual processing within the occipital cortex depends on eye movement type and is not solely related to the processing of stimulus features. This interpretation is in accordance with the identification of binocular neurons within V2, as already reported by Hubel and Wiesel (1970) [61] and Chen et al. (2008) [62]. Larger activity for combined convergence compared also to combined divergence may reflect the higher number of near cells than far cells within this area, however, according to our knowledge, there is no evidence to support this hypothesis. Alternatively, the increased activity may also be viewed as an attentional modulation of the P1 component (e.g., see Van der Lubbe and Woestenburg, 1997 [63]), which would suggest that more attention is needed for combined convergences than for combined divergences and pure saccades. To sum up, the activity observed within the occipital cortex in our study seems to reflect the further processing of specific stimulus features that crucially depends on the type of eye movement that has to be performed.

For FEF, the largest activity was observed for both vergence conditions. Therefore, we think that this pattern of activity reflects a process related to depth cues, i.e. retinal disparity. The engagement of frontal areas in vergence eye movements was also observed and emphasized by Gamlin and Yoon [64]. They found that near and far response neurons, stimulated by crossed and uncrossed disparity respectively, are present in FEF. Additionally, Ferraina et al. [65] showed that many FEF neurons are sensitive to retinal disparity. She also suggested that FEF, beyond the detection of retinal disparity, may be engaged in shifting fixation to stimuli in three-dimensions. The observed anatomical connections seem also in line with the idea that FEF is related to vergence eye movements, since connections have been found with areas that are sensitive to retinal disparity, like the primary visual cortex [25], a middle temporal area [66], medial temporal superior area [67] and the nucleus reticularis tegmenti pontis [30] in the brainstem.

As indicated above, we think that the third regional source that we localized in an anterior frontal area, is possibly a residual eye movement artifact. We observed more activity for this source when preparing complex eye movements, which might be related to a kind of tremor generated by the eyes that is triggered by depth cues. Alternatively, this prefrontal activity may reflect the activity of a “compensatory mechanism” that was initially observed in the elderly, as larger involvement of prefrontal cortex was observed in both simple and complex tasks [68], which might enable the maintenance of a high task accuracy. Interestingly, a recent study by Berchicci et al. (2012) revealed that in younger adults (close in age to our participants) increased prefrontal activity was observed in a more challenging task [69], which requires additional cognitive processes. Nevertheless, since there is no report in the literature that relates this area with the preparation and/or execution of reflexive eye movements, this alternative interpretation remains tentative.

One might argue that the possible differences between convergences and divergences in our study are possibly due to small differences between the near and far LEDs. To examine this possibility, we compared stimulus-locked source activities for saccades towards far and near locations. Results showed similar activities for both distances. As a consequence, the observed differences in the source activities between convergences and divergences cannot be ascribed to discrepancies between near and far LEDs.

Finally, we were surprised not to see any engagement of the parietal lobe. The posterior parietal cortex (PPC) is known to take part in the process of reading [70], shifting spatial and temporal attention with regard to saccades [71], and part of PPC—PEF is thought to disengage fixation and trigger reflexive saccades [13]. Although parietal areas are also thought to be important for vergences [72, 73, 74, 75], our results did not identify a source within the parietal lobe. As our model was especially built on combined convergences, this lack of support for a parietal source may imply that this area is related rather to saccades than to vergences, and it may not have revealed processes that are strongly related to saccades. This issue will have to be explored in future studies.

Interestingly, Berchicci et al. also used the BESA method to model the intracranial sources of cortical potentials related to eye movements [76]. They investigated volitional (self-paced) saccades, which cannot be directly compared with reflexive eye movements. In line with our results, they also observed active sources before eye movement onset that were localized within FEF and occipital areas. However, they also observed activity within the intra-parietal sulcus (IPS) and SEF. The absence of these sources in our study may be due to the short time interval between stimulus onset and the onset of the eye movement that could be used for the analyses, since the self-paced saccade paradigm allows investigating a much longer time interval before eye movement onset (about 2,500 ms).

According to our knowledge, this is the first study using a source analysis on ERPs to specify the cortical areas related to the execution of exogenous saccades, combined divergences and convergences. This approach enabled us to specify the relevant neural correlates of eye movements better than approaches that are solely based on ERPs. Furthermore, it allowed us to describe the involvement of these areas with a high temporal resolution, which provides important information about the interplay and role of these areas. This led us to conclude that the identified cortical areas seem more involved with sensory aspects related to the execution of the different eye movement types and not so much their execution. We hypothesize that motor aspects responsible for final execution of the movement are controlled by subcortical areas.

## Supporting information

S1 Table Clinical parameters of optometric examination for heterophoria, positive (base-out) and negative (base in) fusional range, the break and recovery point of near point of convergence. In heterophoria measurement + indicates esophoria, whereas–exophoria.
(DOCX)
