# Supplementary material for: The engagement of cortical areas preceding exogenous vergence eye movements
Source: PLoS One. 2018 Jun 8;13(6):e0198405. doi: 10.1371/journal.pone.0198405 (PMC5993318; doi:10.1371/journal.pone.0198405)
Supplement: S1 Table — In heterophoria measurement + indicates esophoria, whereas–exophoria. (DOCX) [file pone.0198405.s001.docx]

**Table. Clinical parameters of optometric examination for heterophoria, positive (base-out) and negative (base in) fusional range, the break and recovery point of near point of convergence.** In heterophoria measurement + indicates esophoria, whereas – exophoria.

| Parameter | Subject | | | | | | | | | | | | | |  |
| --- | --- | --- | --- | --- | --- | --- | --- | --- | --- | --- | --- | --- | --- | --- | --- |
|  | **S1** | **S2** | **S3** | **S4** | **S5** | **S6** | **S7** | **S8** | **S9** | **S10** | **S11** | **S12** | **S13** | **S14** | |
| Heterophoria at far (prdptr) | -1 | 0 | 0 | -1 | 0 | -2 | -1 | 0 | 0 | 0 | 0 | 0 | -1 | 0 | |
| Positive fusional range at far (prdptr) | 12 | 20 | 20 | 20 | 25 | 21 | 25 | 24 | 14 | 12 | 23 | 14 | 14 | 18 | |
| Negative fusional range at far (prdptr) | 9 | 12 | 8 | 8 | 9 | 10 | 9 | 8 | 8 | 6 | 9 | 7 | 8 | 8 | |
| Heterophoria at near (prdptr) | -1 | -2 | -6 | -2 | -2 | -6 | -4 | -1 | 1 | -1 | -4 | -4 | -2 | 0 | |
| Positive fusional range at near (prdptr) | 20 | 25 | 35 | 30 | 35 | 28 | 35 | 30 | 20 | 22 | 25 | 19 | 18 | 30 | |
| Negative fusional range at near (prdptr) | 16 | 18 | 25 | 14 | 18 | 14 | 16 | 12 | 14 | 14 | 18 | 16 | 14 | 16 | |
| Near point of convergence – break (cm) | 1 | 4 | 3 | 1 | 4 | 5 | 4 | 2 | 2 | 4 | 5 | 5 | 6 | 1 | |
| Near point of convergence – recovery (cm) | 2 | 5 | 5 | 2 | 6 | 7 | 5 | 3 | 4 | 6 | 7 | 6 | 7 | 2 | |
